# Supplementary material for: Massively parallel immunopeptidome by DNA sequencing provides insights into cancer antigen presentation
Source: Nat Genet. 2025 Jul 28;57(8):2062–73. doi: 10.1038/s41588-025-02268-1 (PMC12339365; doi:10.1038/s41588-025-02268-1)

nature genetics

Supplementary information https://doi.org/10.1038/s41588-025-02268-1

Massively parallel immunopeptidome by DNA sequencing provides insights into cancer antigen presentation

In the format provided by the authors and unedited

Supplementary Information

List of Supplementary Tables

Supplementary Table 1. Positive and negative peptides used for Figure 1

NLVPMVATV	A*0201	pp65-positive peptide
SRYWAIRTR	A*0201	No binding peptide, flu
YTAVVPLVY	A1	pos
CRAPRKKGC	A1	neg
AIFQSSMTK	A1	neg
ATDALMTGY	A1	pos
RPRGEVRFL	B7	pos
IPQCRLTPL	B7	pos
YTAVVPLVY	B7	neg
CRAPRKKGC	B7	neg
YFDPANGKF	C4	pos
NYDQTVSRM	C4	pos
AQEDGAPVS	C4	neg
ESNGNRLIA	C4	neg

Supplementary Table 2. List of peptides with different affinity from IEDB for Figure 1.

peptide	Affinity (IEDB)
FTWQHNYYL	0.94
YMKPGSSPL	0.87
ILYNEYNFV	0.76
ALMEVTHVL	0.7
FLKDVMESM	0.64
RVYAELAAL	0.58
RVRQAWDTL	0.38
YPASLHKFF	0.31
GPAGYTAAL	0.08
RIYRKGNPL	0.37

Supplementary Table 3. List of SARS-CoV2 strain and mutations, related to Figure 3.

strain name	Mutations *
19B	L452R,H655Y,N501T,A653V,D796Y,L18F,G1219V,HV69X
20A	A222V,D614G,S477N
A23	F157L,P681H,R102I,Q613H
Alpha	P681H,S235F,G204R,S982A,A570D,D3L,Y144X,T716I,D614G,HV69X,R203K,D1118H,N501Y
B1	P681H,Y453F,T95I,D796H,Y144X,E484K,D614G
Beta	D80A,E484K,A701V,D215G,L18F,D614G,K417N,T205I,N501Y
Delta	L452R,D950N,EFR156G,D377Y,T19R,T478K,D63G,P13L,D614G,P681R,R203M
Epsilon	L452R,D614G,S13I,W152C
Eta	E484K,D3Y,A67V,S2X,Q677H,Y144X,F888L,D614G,HV69X,A12G,T205I
Gamma	D138Y,E484K,T20N,G204R,K417T,P26S,T1027I,R190S,P80R,V1176F,L18F,H655Y,D614G,R203K,K417N,N501Y
Iota	T95I,P199L,A701V,E484K,L5F,D253G,D614G,M234I,S477N
Kappa	L452R,Q1071H,E154K,D377Y,G142D,D614G,E484Q,P681R,R203M
Lambda	L452R,F490S,G204R,T76I,G75V,G214C,D253N,RSYLTPG246X,P13L,D614G,R203K,T859N
Mu	P681H,D950N,T95I,E484K,D614G,R346K,T205I,N501Y
Omicron	E484A,N764K,G446S,T478K,K417N,T547K,GVYY142D,NL211I,N969K,N856K,D614G,ERS31X,G496S,P681H,H655Y,Y505H,S373P,Q954H,D796Y,S371L,HV69X,R203K,S477N,N440K,T95I,G204R,S375F,A67V,G339D,Q493R,N679K,L981F,N501Y
Theta	P681H, E484K, G204R, H1101Y, V1176F, E1092K, D614G, R203K, N501Y
Zeta	D614G, E484K, V1176F

* X denotes deletion

Supplementary Table 4. List of oncogenic point mutations and gene fusion junctions. Related to Figure 4-5. See separate Supplementary Information Excel file

Supplementary Table 5. List of peptides sequences, primers

name	sequence	note
GH_sp_nested1_fw	ccaccatggcgacgggttca	PCR1: Amplify pooled peptides with a single allele
huB2m_PCR_mid_rev	gtacaagagatagaaagaccagtccttgctga	
P7_Tru_GH_HLA_fw	GTGACTGGAGTTCAGACGTGTGCTCTTCCGATCTggttacaggagggctcggca	PCR2 to add Illumina adapters
P5_TruS_b2m_rev	ACACTCTTTCCCTACACGACGCTCTTCCGATCTgaaagacaagtctgaatgctccact	
GH_sp_nested1_fw	ccaccatggcgacgggttca	PCR1: Amplify pooled peptides with barcoded HLAs mix/pool (combinatorial pool)
10xTSOlink_b2m_HLA_rev	cctcctccgTTTCTTATATGGGaacct	
P5_TruS_bcB2m_1rev	ACACTCTTTCCCTACACGACGCTCTTCCGATCTcacccatgtctcgatcccact	With P7 primer above used in PCR2 for combinatorial pool
amp_spHLA_fw	Gttacaggagggctcggca	Amplify peptide oligo pool for cloning
amp_lkHLA_rev	CCggaccctccgcatcc	

Supplementary Table 6. List of spike-in peptides for Figure 4-5
See separate Supplementary Information Excel file

Supplementary Table 7. List of known antigens in oncogene pools for Figure 4-5
See separate Supplementary Information Excel file

Supplementary Table 8. List of peptides nominated by ESCAPE-seq used for validation assay for Figure 6.

Label	Name	Sequence
p1	BCR_ABL1_6	KQSSKALQR
p2	CCDC6_RET_7	VTIEDPKWE
p3	CRTC1_MAML1_8	RLQGSLKRK
p4	EML4_ALK_10	LYRRKHQEL
p5	ETV6_NTRK_6	RIADVQHIK
p6	EWSR1_WT1_3	SSYGQQSEK
p7	EWSR1_FLI1_4	YGQQSSLLA
p8	EWSR1_FLI1_5	GQQSSLLAY
p9	KIAA_BRAF_4	AYIGCPDLI
p10	NCOA4_RET_9	SQEDPKWEF

p11	NPM1_ALK_2	HISGQHLVV
p12	SS18_SSX2_5	YGYDQIMPK
p13	TCF3_PBX1_6	PDSYSVLSI
p14	EZH2Y646S_4	FISESCGEI
p15	HRASG13R_8	RVGKSALTI
p16	HRASG13V_8	VVGKSALTI
p17	EGFRT790M_0	LTSTVQLIM
p18	EGFRT790M_1	TSTVQLIMQ
p19	EGFRT790M_2	STVQLIMQL
p20	EGFRT790M_3	TVQLIMQLM
p21	EGFRT790M_4	VQLIMQLMP
p22	EGFRT790M_5	QLIMQLMPF
p23	EGFRT790M_6	LIMQLMPFG
p24	EGFRT790M_7	IMQLMPFGC
p25	EGFRT790M_8	MQLMPFGCL

Supplementary Note 1, IEDB data and population wide coverage.
We compared the frequency of each allele across the population to the number of IC50 binding affinity measurements that currently exist for this allele. **Fig. SN-1a** below showed each HLA alleles' number of data in IEDB vs its frequency in population (where world population data was downloaded from IEDB: http://tools.iedb.org/population/download/). We saw that HLA-A alleles are in the upper left, indicating a higher ratio of binding affinity measurements over frequency whereas there are many HLA-C alleles (green) at the bottom middle, showing that there are many HLA-C alleles that are quite common and yet have very little existing binding affinity measurements. It is worth noting that this is limited to IC50 binding affinity measurements, although it has been observed that datasets derived from Mass Spectrometry or other peptide presentation assays also underrepresent HLA-C[13].

To understand the consequences of this, in **Fig. SN-1b**, we calculated how many HLA alleles from each gene we would need to get measurements for in order to have good coverage across the population. Specifically, we sampled increasing numbers of alleles from each gene. Then, for each person in our synthetic population, we can check whether at least 1 of their assigned alleles for that gene is in our dataset. As with other simulations, this is then repeated with 10 different synthetic populations for confidence intervals. We see that HLA-C alleles are consistently the most efficient at covering the population and can cover 50% of the population in less than half the alleles it would take HLA-B to do the same.

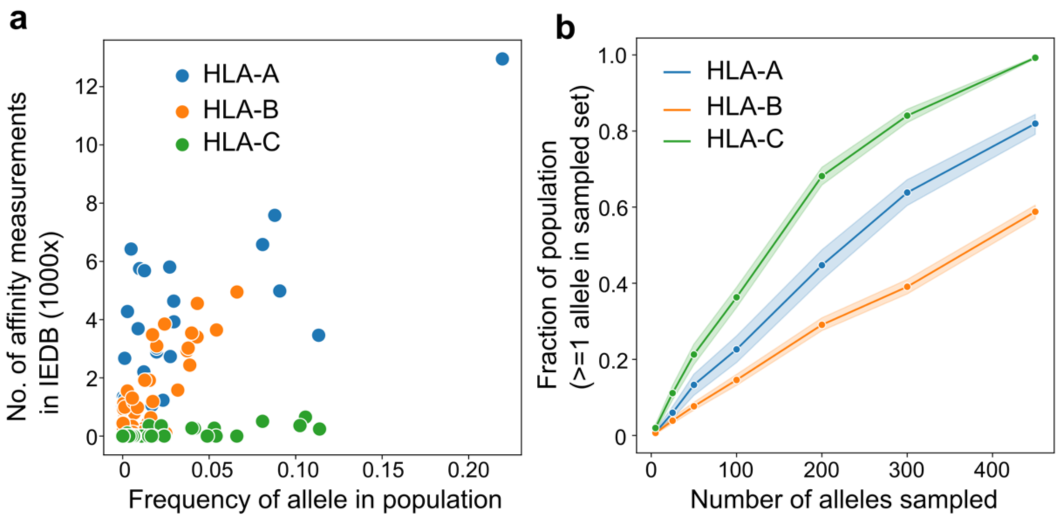

Fig. SN-1
This is not surprising as the population-level statistics in IEDB only has metrics for ~500 HLA-C alleles as compared to 1200 HLA-B alleles, so it makes sense each HLA-C allele cover more of the population.

Supplementary Note 2, choice of E-score threshold (cutoff).
The E-score essentially indicates the ranking of peptide presentability or binding strength to a specific HLA allele. Using HLA-A*02:01 data as an example (Fig. SN-2), we binned all A*02 peptides based on their E-scores from ESCAPE-seq into three categories: **high** (E-score > 6), **medium** (E-score between 3.8–6), and **low** (E-score between 2.9–3.8). This binning approach was designed to ensure a roughly equal number of peptides in each category. A comparison of the motifs across these bins reveals a progressive loss of amino acid preferences at anchor positions, corresponding to a decrease in peptide binding affinity. This suggests that peptide binding affinity to HLA alleles likely follows a continuous distribution, decreasing to a point where peptides are no longer considered binders.

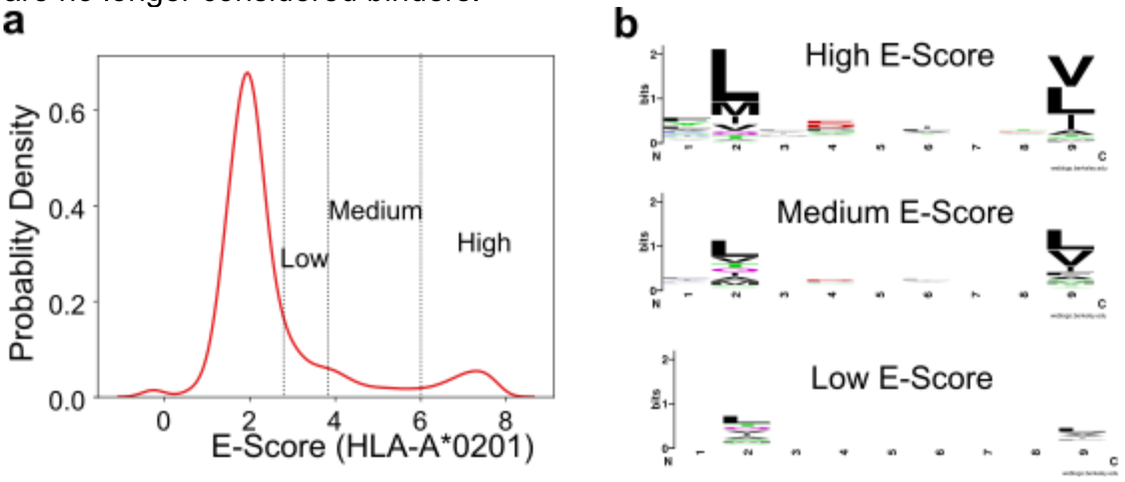

Fig. SN-2, E-score distribution for HLA-A*02:01 (**a**) and peptide motif patten (**b**) based on different E-score bins as illustrated in **(a).**

However it is often useful to define a cutoff value to distinguish presented peptides from non-presented ones, in ESCAPE-seq, we aimed to use a universal E-score cutoff across all alleles. For this purpose, E-scores were normalized to ensure that the background peak was centered at 2 (Methods). Generally, ESCAPE-seq experiments on single alleles exhibit less noise compared to combinatorial ESCAPE-seq experiments, which involve a larger number of peptide-HLA allele pairs. Based on this, we applied an approximate cutoff of **~3.2** for single alleles and **~3.8–4.0** for most alleles in combinatorial ESCAPE-seq.

However, we observed that while most HLA class I alleles display similar E-score distribution profiles (**Fig. SN-3a**), characterized by a dominant background peak, some alleles exhibit additional peaks in the middle E-score range or broader background peaks (marked by arrows in **Fig. SN-3b**). For these specific alleles, we increased the cutoff values to between **4.5–5.5**, based on their E-score distributions (as illustrated in **Fig. SN-3**b).

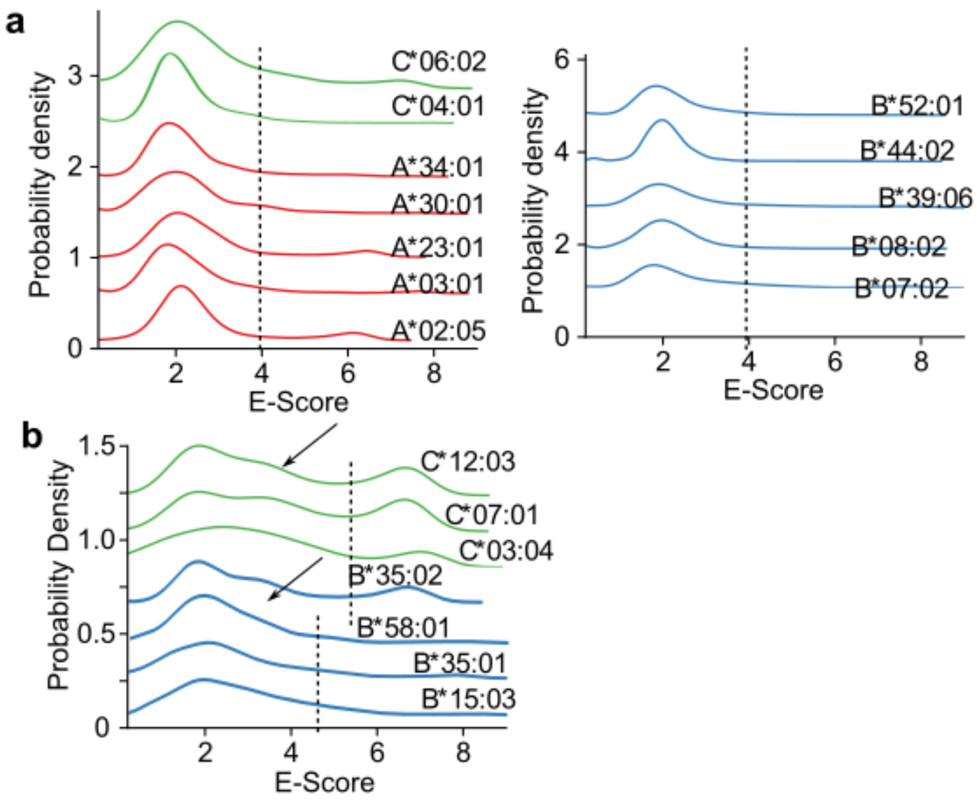

Fig. SN-3, Examples of E-score distributions for multiple alleles.

Supplementary Figure 1. Diagrams of FACS gating strategies

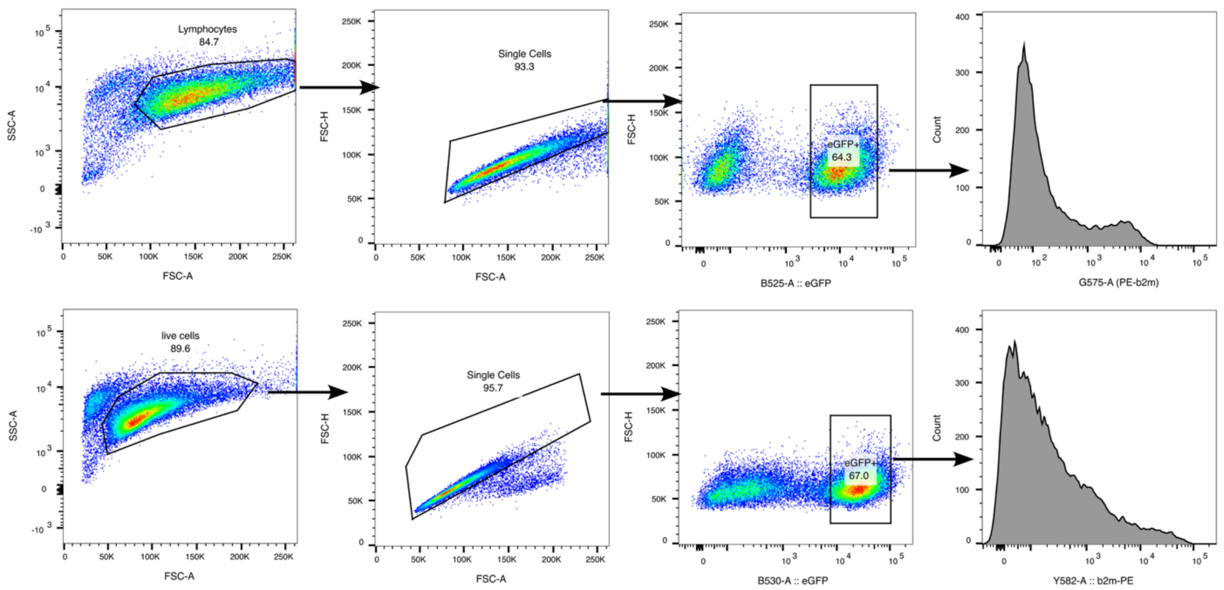

Supplement: Supplementary file 1 — Supplementary Tables 1–3, 5 and 8, Notes 1 and 2 and Fig. 1. [file 41588_2025_2268_MOESM1_ESM.pdf]
