# Supplementary material for: Massively parallel immunopeptidome by DNA sequencing provides insights into cancer antigen presentation
Source: Nat Genet. 2025 Jul 28;57(8):2062–73. doi: 10.1038/s41588-025-02268-1 (PMC12339365; doi:10.1038/s41588-025-02268-1)
Supplement: Supplementary file 2 — Reporting Summary [file 41588_2025_2268_MOESM2_ESM.pdf]

nature portfolio

Corresponding author(s): Chang

Last updated by author(s): 2025/06/16

Reporting Summary

Nature Portfolio wishes to improve the reproducibility of the work that we publish. This form provides structure for consistency and transparency in reporting. For further information on Nature Portfolio policies, see our Editorial Policies and the Editorial Policy Checklist.

Statistics

For all statistical analyses, confirm that the following items are present in the figure legend, table legend, main text, or Methods section.

n/a	Confirmed	
☐	☒	The exact sample size (*n*) for each experimental group/condition, given as a discrete number and unit of measurement
☐	☒	A statement on whether measurements were taken from distinct samples or whether the same sample was measured repeatedly
☐	☒	The statistical test(s) used AND whether they are one- or two-sided<br>*Only common tests should be described solely by name; describe more complex techniques in the Methods section.*
☒	☐	A description of all covariates tested
☒	☐	A description of any assumptions or corrections, such as tests of normality and adjustment for multiple comparisons
☐	☒	A full description of the statistical parameters including central tendency (e.g. means) or other basic estimates (e.g. regression coefficient) AND variation (e.g. standard deviation) or associated estimates of uncertainty (e.g. confidence intervals)
☒	☐	For null hypothesis testing, the test statistic (e.g. *F*, *t*, *r*) with confidence intervals, effect sizes, degrees of freedom and *P* value noted<br>*Give P values as exact values whenever suitable.*
☒	☐	For Bayesian analysis, information on the choice of priors and Markov chain Monte Carlo settings
☒	☐	For hierarchical and complex designs, identification of the appropriate level for tests and full reporting of outcomes
☐	☒	Estimates of effect sizes (e.g. Cohen's *d*, Pearson's *r*), indicating how they were calculated

Our web collection on statistics for biologists contains articles on many of the points above.

Software and code

Policy information about availability of computer code

Data collection	Data collection was described in the Method section. In particular, the flow data was acquired with commercial Attune software (Thermofisher). Sequencing data was acquired with commercial Illumina Sequencer (Nextseq 550 or Novaseq). FACS data was collected using BD Aria.
Data analysis	Flow data were analyzed with commercial software Flowjo (v10.10). Sequence data were analyzed with custom python script (py2.7). Some very common python packages were used for analysis, including Biopython (v1.7), Pandas(0.24), and Matplotlib (v2.2). Codes scripts are included in Supplementary data files.

For manuscripts utilizing custom algorithms or software that are central to the research but not yet described in published literature, software must be made available to editors and reviewers. We strongly encourage code deposition in a community repository (e.g. GitHub). See the Nature Portfolio guidelines for submitting code & software for further information.

Data

Policy information about availability of data

All manuscripts must include a data availability statement. This statement should provide the following information, where applicable:

- Accession codes, unique identifiers, or web links for publicly available datasets
- A description of any restrictions on data availability
- For clinical datasets or third party data, please ensure that the statement adheres to our policy

All sequencing files were deposited to SRA. Accession number was provided. Other data, materials and analysis scripts are available upon request. Details were described in Data availability statement

Research involving human participants, their data, or biological material

Policy information about studies with human participants or human data. See also policy information about sex, gender (identity/presentation), and sexual orientation and race, ethnicity and racism.

Reporting on sex and gender	n/a
Reporting on race, ethnicity, or other socially relevant groupings	n/a
Population characteristics	n/a
Recruitment	n/a
Ethics oversight	n/a

Note that full information on the approval of the study protocol must also be provided in the manuscript.

Field-specific reporting

Please select the one below that is the best fit for your research. If you are not sure, read the appropriate sections before making your selection.

☒ Life sciences ☐ Behavioural & social sciences ☐ Ecological, evolutionary & environmental sciences

For a reference copy of the document with all sections, see nature.com/documents/nr-reporting-summary-flat.pdf

Life sciences study design

All studies must disclose on these points even when the disclosure is negative.

Sample size	For individual validation, we perform at least 2 biological replicates to check the variation, and do more replicates when see a larger variation. In the pooled screening, we used cell number of >1000 times of the input library size in order to cover the distribution of input variants, on the range of 5million to 100million cells per screening, and replicated twice. We included Statistics and reproducibility statement.
Data exclusions	in the screening, each candidate (a peptide-HLA pair), if its read is low (e.g. <40), is excluded for downstream analysis. This is due to the fact that when read count is low, there is more error in all measurements, thus the result will not be reliable. So it is better to exclude those data point.
Replication	We performed at least 2 biological replicates in all our experiments to check the variation. We did more replicates when observing a larger variation (e.g. Coefficient of Variation> 10%). all replications are highly correlated and reproducible.
Randomization	We only used cells in our assay here instead of animals. We take cells randomly but with a fix number depending on experimental purpose.
Blinding	Blinding maybe is not applicable to our screening case here. But the investigator did not have any prior knowledge about the hits (i.e. peptide-HLA pairs) until the data were analyzed at the end. So in this persepctive, investigators were blinding to samples/results.

Reporting for specific materials, systems and methods

We require information from authors about some types of materials, experimental systems and methods used in many studies. Here, indicate whether each material, system or method listed is relevant to your study. If you are not sure if a list item applies to your research, read the appropriate section before selecting a response.

Materials & experimental systems

n/a	Involved in the study
☐	☒ Antibodies
☐	☒ Eukaryotic cell lines
☒	☐ Palaeontology and archaeology
☒	☐ Animals and other organisms
☒	☐ Clinical data
☒	☐ Dual use research of concern
☒	☐ Plants

Methods

n/a	Involved in the study
☒	☐ ChIP-seq
☐	☒ Flow cytometry
☒	☐ MRI-based neuroimaging

Antibodies

Antibodies used: PE anti-b2m (clone 2M2); Pe-Cy7 anti human HLA-A2 (clone BB7.2),CD3 (clone SK7, FITC), CD4 (clone RPA-T4, BV785) and CD8a (clone RPA-T8, APC), IFN-g (clone B27, PE) and TNF-a (clone Mab11, PE/Cy7) from Biolegend. anti-CD28 (BD Biosciences) and anti-CD49d (BD Biosciences). Usage and dilution follows manufacturer's protocol. for screening, we use 2ul (0.2mg/ml) of b2m antibody per 1M cells.

Validation: We follow manufacturer's protocol and previous published amount to stain the cells. specifically antibodies are from Biolegend and BD with their validation information at https://www.biolegend.com/fr-fr/bio-bits/highly-specific-validated-antibodies and https://www.bdbiosciences.com/en-us/products/reagents/flow-cytometry-reagents/research-reagents/quality-and-reproducibility

Eukaryotic cell lines

Policy information about cell lines and Sex and Gender in Research

Cell line source(s): HEK293T cells from ATCC, with its endogenous HLA knocked-out, PBMC donor samples purchased from https://immunospot.com/

Authentication: HEK293T cells were purchased from and validated by ATCC.

Mycoplasma contamination: Mycoplasma test was done with Lonza MycoAlert kit and/or InvivoGene MycoStrips. tested negative.

Commonly misidentified lines (See ICLAC register): no commonly misidentified cell lines were used in the study

Plants

Seed stocks: n/a

Novel plant genotypes: n/a

Authentication: n/a

Flow Cytometry

Plots

Confirm that:

☒ The axis labels state the marker and fluorochrome used (e.g. CD4-FITC).

☒ The axis scales are clearly visible. Include numbers along axes only for bottom left plot of group (a 'group' is an analysis of identical markers).

☒ All plots are contour plots with outliers or pseudocolor plots.

☒ A numerical value for number of cells or percentage (with statistics) is provided.

Methodology

Sample preparation: HEK293T with endogenous HLA knock-out cells were used to stain with specific antibodies. As described in the Method, cells

Sample preparation	were trypsinized, incubated after wash before staining of antibody at 4c for 30min; then wash and run flow cytometry.
Instrument	We used Attune flowcytometry (Thermofisher) for flow analysis only; and used BD FACSAria (BD biosciences) for cell sorting.
Software	For Attune, the software coming along with the instrument were used for data collection. BD Diva software was used for FACS. Afterward, Flowjo was used for analysis
Cell population abundance	In our screening, we aimed to have live cell count at least 1000x of input diversity; this strategy was shown by previous publications to be able to reliably capture the distribution of input variants well for screening experiments. Then we sorted the cells in purity mode. For example, with 75000 variants in our oncogene pHLA pool, we sorted over 100M live cells per replicates. The last gated cell population were sorted into 4 bins with different staining intensity.
Gating strategy	The flow plots were first gated with SSC vs FSC to get live cell population; next gated on single cells to remove doublets or aggregate with FSC-H vs FSC-A plot. Afterward, cells with successfully transduced pHLA variant was gated based on eGFP marker; and finally gated with the PE-anti b2m staining and sort the cell population into 4 bins. To distinguish positive and negative, a negative control either wihout eGFP or without staining, or with antibody staining but no surface expression, were used. Since our staining or eGFP signals were very bright, the positive and negative were separately quite far between each other and easy to tell.

☒ Tick this box to confirm that a figure exemplifying the gating strategy is provided in the Supplementary Information.
