# Supplementary material for: Massively parallel immunopeptidome by DNA sequencing provides insights into cancer antigen presentation
Source: Nat Genet. 2025 Jul 28;57(8):2062–73. doi: 10.1038/s41588-025-02268-1 (PMC12339365; doi:10.1038/s41588-025-02268-1)
Supplement: Supplementary file 4 — Methods and QC. [file 41588_2025_2268_MOESM4_ESM.pdf]

MS Bioworks
Protein Mass Spectrometry Services

3950 Varsity Drive
Ann Arbor, MI 48108
734-929-5083
www.msbioworks.com
info@msbioworks.com

Project Report

Information

Client:	Quanming Shi
Institute:	Stanford University
Project Number:	MSB-11814
Date Submitted:	July 2nd, 2024
Date Completed:	August 7th, 2024

Samples

Client identifier	MSB identifier	Notes
HEK293T	71837	400M

Objective

Class I MHC peptide analysis from human cell samples: inject 50% in duplicate with a 2h gradient and dual fragmentation (CID and EThcD).

Experimental Methods

Sample Preparation

Peptides (100%) were desalted using solid-phase extraction (SPE) with Wates µHLB C18 plate. Peptides were loaded directly and eluted using 30/70 acetonitrile/water (0.1% TFA). Eluted peptides were lyophilized and reconstituted in 0.1% TFA.

Mass Spectrometry

Peptides (50%) were analyzed in analytical duplicate by nano LC/MS/MS using a Waters NanoAcquity system interfaced to a ThermoFisher Fusion Lumos mass spectrometer. Peptides were loaded on a trapping column and eluted over a 75µm analytical column at 350nL/min; both columns were packed with Luna C18 resin (Phenomenex). A 2h gradient was employed. The mass spectrometer was operated using a custom data-dependent method, with MS performed in the Orbitrap at 60,000 FWHM resolution and sequential

MS/MS performed using high resolution CID and EThcD in the Orbitrap at 15,000 FWHM resolution. All MS data were acquired from m/z 300-1600. A 3s cycle time was employed for all steps.

Data Processing

Raw files were searched using a local copy of PEAKS with the following parameters:

Enzyme: None
Database: Swissprot Human + target sequences
Fixed modification: None
Variable modifications: Oxidation (M), Acetyl (Protein N-term), Carbamidomethyl (C)
Mass values: Monoisotopic
Peptide Mass Tolerance: 10 ppm
Fragment Mass Tolerance: 0.02 Da
Max Missed Cleavages: N/A
PSM FDR: 1%
Chimeric peptide: TRUE

The peptide.csv file was exported.

Results

A total of 843 peptides were detected at the 1% PSM FDR (based on forward/decoy database searching). Please refer to the accompanying Excel file for the full list of peptides and associated information. The Excel file contains 2 worksheets:

Worksheet 1 (PEAKS DB) contains the full list of peptides, their peak intensity and total features. An overview of peptide totals is shown here:

**MSB Identifier**	**71837A**	**71837B**
Total Peptides	605	746
Total Intensity	9.1E+08	1.2E+09

A histogram distribution of peptide length is shown below:

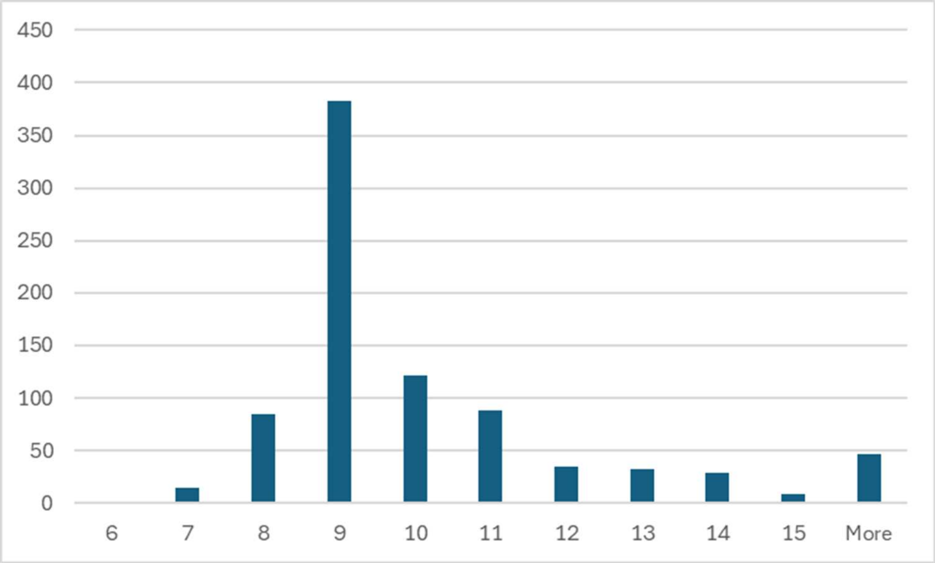

Motif Analysis

Peptides >12AA in length were omitted from the motif analysis.

Known (top) and experimental (bottom) motifs for each allele are presented below along with the number of matched peptides:

C*03:04

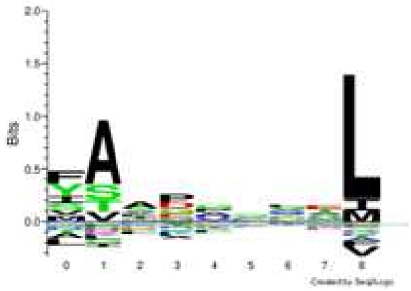

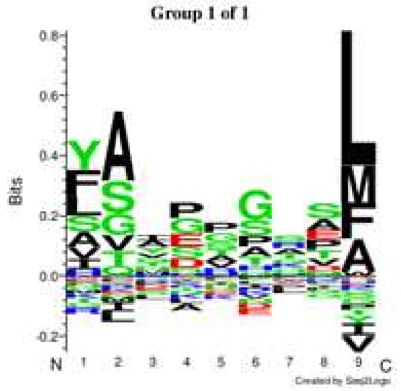

602 peptides

Worksheet 2 (Target) contains the target peptide matches.
